# Repeatability of selective laser trabeculoplasty for open-angle glaucoma

**DOI:** 10.1186/s12886-016-0299-9

**Published:** 2016-07-28

**Authors:** Brian A. Francis, Nils Loewen, Bryan Hong, Laurie Dustin, Kevin Kaplowitz, Robert Kinast, Jason Bacharach, Sunita Radhakrishnan, Andrew Iwach, Lidiya Rudavska, Parul Ichhpujani, L. Jay Katz

**Affiliations:** 1 Department of Ophthalmology, David Geffen School of Medicine at UCLA, Doheny Eye Institute, 800 Fairmount Avenue, Suite 215, Pasadena, Los Angeles, CA 91105 USA; 2 Department of Ophthalmology, University of Pittsburgh, School of Medicine, Pittsburgh, PA USA; 3 Department of Ophthalmology, Thomas Jefferson School of Medicine, Wills Eye Institute, Philadelphia, PA USA; 4 Department of Preventive Medicine, Keck School of Medicine of the University of Southern California, Los Angeles, CA USA; 5 Department of Ophthalmology, Stony Brook University, Stony Brook, NY USA; 6 North Bay Eye Associates, Sonoma County, CA USA; 7 Glaucoma Center of San Francisco, San Francisco, CA USA; 8 Research and Education Group, San Francisco, CA USA; 9 Wills Eye Hospital, Thomas Jefferson University Medical School, Philadelphia, PA USA

**Keywords:** Selective, Laser, Trabeculoplasty, Repeat, Repeatability, Glaucoma

## Abstract

**Background:**

To analyze the results of repeat selective laser trabeculoplasty (SLT).

**Methods:**

Inclusion criteria: participants with primary or secondary open-angle glaucoma (excluding uveitic) who had undergone SLT 360° (SLT 1) with diminution of response over time followed by repeat SLT 360° (SLT 2). Six months of follow-up were required and at least 6 months in between SLT 1 and 2. The main outcome measures were IOP reduction at 6 and 12 months and a comparison of the response between SLT 1 and 2.

**Results:**

One hundred thirty-seven patients met the inclusion criteria. If only one eye had repeat treatment, that eye was chosen; if both eyes qualified, one was chosen at random. The baseline intraocular pressure (IOP) for SLT 1 = 20.3+/− 5.2 mmHg and SLT 2 = 19.4 +/− 5.0 was reduced to 16.4 +/− 3.9 and 16.7 +/− 4.7 at 1 year, respectively (*p* < .001). Medication use was not significantly changed, and was 2.2 +/− 1.2 at baseline for SLT 1 and 2.1 +/− 1.3 for SLT 2, and at 1 year was 1.9 +/− 1.3 and 2.2 +/− 1.2, respectively. A subanalysis of 62 patients matched for equivalent baselines showed a baseline IOP = 18.7 +/− 3.8 for SLT 1 and 18.7 +/− 3.5 for SLT 2, reduced to 16.0 +/− 4.3 and 15.3 +/− 3.8 at 1 year (*p* < .001).

**Conclusion:**

Repeat SLT laser (360-degree treatment, followed by a loss of effect over time, then a second 360-degree treatment) in this population resulted in IOP lowering similar to that of the initial treatment.

## Background

Laser trabeculoplasty has been employed as initial, adjunct or replacement therapy to lower intraocular pressure (IOP) in patients with open-angle glaucoma (OAG). The original procedure was described using argon laser (major peaks at 488 and 514 nm). Laser trabeculoplasty has been used to successfully lower the IOP and slow visual field progression in several multicenter randomized trials, notably the Early Manifest Glaucoma Trial [1] and the Advanced Glaucoma Intervention Study [2]. The Glaucoma Laser Trial showed that in patients with newly diagnosed open-angle glaucoma, argon laser trabeculoplasty (ALT) was at least as effective as initial treatment with timolol maleate 0.5 %, even after 7 years [3, 4]. However, ALT produces significant tissue disruption and coagulative damage to the trabecular meshwork, possibly contributing to the limited success rates reported after retreatment [5, 6]. Indeed, repeated treatment of the angle with argon laser will eventually lead to synechial angle closure and a decrease in outflow facility. Some very preliminary evidence suggested that the inflammation induced by trabeculoplasty could also lead to failure of subsequent trabeculectomy because of increased scarring [7]. This, coupled with the fact that most patients eventually ended up on medications, led to the failure of acceptance of ALT as primary glaucoma therapy. Most physicians in the United States maintained the algorithm of maximum tolerated medications first, then laser trabeculoplasty, and finally filtration surgery.

The treatment algorithm may be changing with the use of selective laser trabeculoplasty (SLT), approved by the FDA in March 2001 for the treatment of OAG. Using the 532 nm, frequency-doubled, Q-switched neodymium-doped yttrium aluminium garnet (Nd:YAG) laser, SLT results in the selective absorption of energy by pigmented cells in the trabecular meshwork and spares adjacent cells and tissues from thermal energy damage [8]. Compared to ALT, each SLT pulse delivers less than 0.1 % total energy and is eight orders of magnitude shorter in duration.

SLT was initially studied as a secondary modality in cases of failed medical therapy or prior ALT [9–12]. More recently, SLT has proven effective as a primary treatment in OAG with minimal side effects or complications [13–16]. It has also been used as a replacement for medical therapy in controlled open-angle glaucoma [17].

Because of the very short pulse duration of SLT (compared to thermal relaxation time of the tissue), the adjacent tissues do not absorb the laser energy, and the spread of heat damage is minimized. The significant decrease in tissue disruption allows for the potential of retreatment with SLT.

The most widely used definition of *repeat* laser trabeculoplasty is applying laser to the same area that has been treated previously. In most cases of SLT, this represents an initial 360-degree treatment followed by a second 360-degree treatment. Due to the coagulative effect of argon laser on the trabecular meshwork, ALT has shown limited success in repeated treatments [18–21].

Retreatment is usually applied when an initial treatment has been successful and the effect diminishes over time. However, it is sometimes applied when the initial response is not enough to reach target IOP levels. In our study, we considered the first scenario. Repeat treatment should be differentiated from augmentation or sequential treatment. Treating 180° followed by laser to the remaining 180° may be termed *augmentation* of treatment. Finally, SLT performed after ALT (or any laser trabeculoplasty procedure followed by treatment with a different laser) should be differentiated as *sequential* treatment with the two laser modalities identified.

We performed a multi-center, retrospective study of repeat SLT in patients who had undergone a 360-degree treatment, followed by a reduction of IOP response over time, and who were then treated with a second laser. To our knowledge, this is only the second report of repeat SLT, but with a much larger cohort and longer follow up. The larger numbers allow for several subanalyses, such as matching for baselines, time between lasers, and type of glaucoma medications, as well as a survival analysis.

## Methods

The inclusion criteria were primary or secondary open-angle glaucoma, age greater than 18 years, and treatment with 360-degree SLT (SLT 1) followed by retreatment of 360-degree SLT (SLT 2) more than 6 months later for decrease of IOP control. A minimum follow-up time of 6 months after SLT 2 was also required for inclusion. Exclusion criteria were uveitic, traumatic, or angle-closure glaucoma, prior glaucoma surgery (except ALT), corneal disease that may affect applanation tonometry, aphakia, and systemic or ocular steroid use.

### Data collection

Demographic information collected included age, gender, race, and glaucoma diagnosis. All patients received a complete ophthalmic exam prior to SLT, including central corneal thickness measurement and optic nerve head exam.

Baseline IOPs for both initial and repeat SLT were defined as the average of two IOPs: the IOP at the visit prior to the SLT and the pre-procedure IOP on the day of laser. Preoperative baseline medication was defined as the number of medications the patient was taking at the time of the pre-operative IOP measurements. Fixed combination medications were counted as two separate glaucoma medications. Postoperative data was collected at 1, 3, 6, and 12 months following both SLT treatments. All IOPs were measured by a certified ophthalmic technician or an ophthalmologist using a calibrated Goldmann applanation tonometer.

### SLT procedure

All initial and repeat SLT procedures were performed by glaucoma specialists at referral glaucoma centers. Prior to initiation of the study, the investigators met to agree on a standardized laser technique to minimize the variation in power, number, and location of laser spots. Eyes were treated with topical tetracaine or proparacaine hydrochloride and all were pretreated with apraclonidine 1.0 % or brimonidine 0.15 % or 0.1 %. The protocol was similar to that initially described by Latina et al. [12]. The Q-switched frequency-doubled 532 nm Nd:YAG laser (Lumenis Selecta SLT Laser, Lumenis Inc., Santa Clara, CA) was used with a wavelength of 532 nm, with a pulse duration of 3 ns. The number of shots ranged from 80 to 132 non-overlapping laser spots over 360° of trabecular meshwork (TM). The energy level for treatment was adjusted between 0.6 and 1.4 mJ to cause formation of small cavitation energy bubbles during at least 50 % of shots.

### Outcome measures

Two definitions of success were used. The first and more stringent is similar to the Tube versus Trabeculectomy Study [22]: the IOP had to be between 5 and 21 mmHg while demonstrating at least a 20 % IOP decrease from baseline and while avoiding the addition of any glaucoma medications or glaucoma procedures. Definition 2 was IOP 5 to 21 mmHg, along with either an IOP lowering by at least 20 %, or reduction of glaucoma medication use and without the need for glaucoma surgery or laser.

Only one eye from each qualifying subject was entered into the analysis. If both eyes qualified, then one was randomly chosen for inclusion. The statistical analysis was performed with SAS V9.2 (SAS Institute, Cary NC), and the accepted level of significance for all tests was α = 0.05. Student’s *t*-test was used to compare mean measures between SLT1 and SLT2 and a paired *t*-test was used to compare mean change from baseline within SLT 1 or 2. Independent sample t-tests and chi-squared tests were used to compare measures between subgroups. Survival analysis was done with the Kaplan-Meier method and compared using the log rank test. The study was powered to detect a difference in mean IOP reduction between the two treatment arms (SLT1 vs SLT2) of 1.7 mmHg or greater, using a power of 80 % and alpha 0.05.

For patients lost to follow-up, IOP measurements and glaucoma medication numbers were included only during the time they were still in the study. Patients were followed and analyzed to the point of failure or until the most recent follow-up recorded.

## Results

One hundred thirty-seven eyes of 137 patients qualified for inclusion in this study. The demographics are presented in Table 1. The mean age was 72.5 +/− 11.9 years, with the majority of patients being Caucasian (76.5 %). Most patients had primary open-angle glaucoma (80.3 %). The number of glaucoma medications at baseline was 2.2 +/− 1.2 for SLT 1 and 2.1 +/− 1.3 for SLT 2. The baseline IOP was 20.3 +/− 5.2 for SLT 1 and 19.4 +/− 5.0 for SLT 2 (p = 0.03). At 6 months, the IOP decreased to 16.3 +/− 4.3 and 16.3 +/− 4.8 and at 12 months to 16.4 +/− 3.9 and 16.7 +/− 4.7, respectively (see Table 2).

**Table 1:** Patient demographics for repeat selective laser trabeculoplasty

Number of eyes	137
Age, mean (standard deviation), range	72.5 (11.9), 41–99
Sex	
Male	38.7 %
Female	61.3 %
Race	
Caucasian	76.5 %
African American	7.4 %
Hispanic	8.8 %
Other	7.4 %
Glaucoma diagnosis	
Primary open-angle glaucoma	80.3 %
Pseudoexfoliation glaucoma	12.4 %
Pigmentary glaucoma	4.4 %
Ocular hypertension	1.5 %
Juvenile open angle glaucoma	1.5 %
Previous argon laser trabeculoplasty	8.8 %

**Table 2:** *Significance of within treatment change, *p* < 0.001. *IOP* intraocular pressure, *SLT* selective laser trabeculoplasty

		*SLT1 Mean (standard deviation)*	*SLT2 Mean (standard deviation)*	*Paired t-test p-value*
IOP	Baseline (*n* = 137)	20.3 (5.2)	19.4 (5.0)	0.03
	6–12 months (*n* = 130,119)	16.3 (4.3)	16.3 (4.8)	0.86
	12–15 months (*n* = 100,99)	16.4 (3.9)	16.7 (4.7)	0.24
IOP	6–12 months	4.1 (4.8)*	2.9 (4.7)*	0.04
decrease	12–15 months	3.5 (4.7)*	2.2 (4.5)*	0.005
IOP %	6–12 months	17.7 (19.8)*	14.7 (24.2)*	0.32
reduction	12–15 months	14.5 (23.4)*	10.9 (22.2)*	0.11

IOP and IOP reduction are in mmHg

**Fig. 1:**
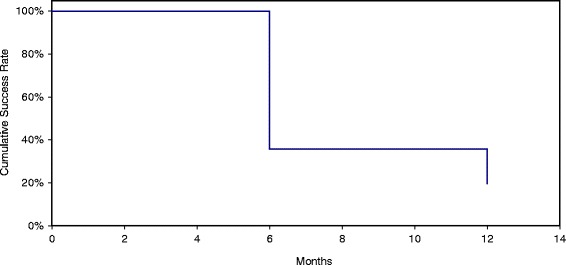
Success of selective laser trabeculoplasty 2 = intraocular pressure (IOP) lowered 20 % or more, and IOP between 5 and 21 mmHg, with no additional glaucoma medications or IOP lowering procedures (Tube versus Trabeculectomy Study criteria)

The success rate for SLT 1 with definition 1 was 55 % at 6 months and 34 % at 12 months (see Fig. 1). Using definition 2, which includes medication reduction, for SLT 1, the rate of success was 65 % at 6 months and 44 % at 12 months. For SLT 2, the success rate with definition 1 was 37 % at 6 months and 19 % at 12 months. For definition number two for SLT 2, the success rate was 48 % at 6 months and 27 % at 12 months.

A sub-analysis was performed with case matching by baseline IOP. This was done due to the lower baseline IOP noted prior to SLT2 compared to prior to SLT1 in the full group analysis. These 62 patients had a baseline IOP prior to SLT 1 that was similar (within 2 mmHg) to the IOP noted prior to SLT 2 (see Table 3). In this subset, the mean IOP prior to SLT 1 was 18.7 +/− 3.8, and the mean prior to SLT 2 was 18.7 +/− 3.5. After 6 months the IOP decreased to 16.0 +/− 4.3 vs 15.3 +/− 3.8 after SLT 2. Twelve months after SLT 1 the mean IOP was 15.8 +/− 3.3 versus 16.6 +/− 4.5 12 months after SLT 2. The success rates were also similar between SLT 1 and 2 in this subgroup. For the first definition, the success of SLT 1 was 43 % at 6 months and 20 % at 12 months, and for SLT 2 it was 44 and 20 %. Using the second definition, the success of SLT 1 was 57 % at 6 months and 33 % at 12 months, and for SLT 2 it was 52 and 28 %, respectively.

**Table 3:** Significance of within treatment change, **p < 0.001, *p < 0.01. *IOP* intraocular pressure, *SLT* selective laser trabeculoplasty

		*SLT1 Mean (standard deviation)*	*SLT2 Mean (standard deviation)*	*Paired t-test p-value*
IOP	Baseline (*n* = 62)	18.7 (3.8)	18.7 (3.5)	0.96
	6–12 months (*n* = 61,52)	16.0 (4.3)	15.3 (3.8)	0.58
	12–15 months (*n* = 49,44)	15.8 (3.3)	16.6 (4.5)	0.33
IOP	6–12 months	2.8 (3.7)**	3.4 (3.6)**	0.64
decrease	12–15 months	2.5 (3.0)**	1.9 (3.5)*	0.38
IOP %	6–12 months	17.7 (19.8)*	14.7 (24.2)*	0.32
reduction	12–15 months	12.7 (15.1)**	11.3 (19.7)*	0.74

Another sub-analysis compared two groups stratified by the amount of time between SLT 1 and 2: less than 1 year versus greater than 1 year (see Table 4). Using the first definition, the success rates for cases where SLT 2 was performed at least 1 year after SLT 1 were 30 % at 6 months and 14 % at 12 months. When SLT 2 was performed within 1 year of SLT 1, the success rates were higher at 59 % at 6 months and 35 % at 12 months (*p* = 0.006). Using definition 2, the success rates were again higher if SLT 2 occurred within 1 year after SLT 1. When SLT 2 was performed 1 year or more after SLT 1, the success rates were 41 % at 6 months and 22 % at 12 months. For less than a 1-year interval, the success was 70 % at 6 months and 45 % at 12 months (*p* = 0.01).

**Table 4:** *SD* standard deviation, *IOP* intraocular pressure, *SLT* selective laser trabeculoplasty. Significance of within treatment change, **p < 0.001, *p < 0.01. *p*-values in the right hand column reflect comparisons for baseline and treated IOP between SLT1 (<12 months in between treatments) and SLT1 (≥12 months between treatments, and SLT2 (<12 months in between treatments) and SLT2 (≥12 months between treatments)

		*Interval between SLT1 and SLT2 ≥ 12 months*			*Interval between SLT1 and SLT2 < 12 months*			
		*SLT1 Mean (sd)*	*SLT2 Mean (sd)*	p SLT1 vs SLT2	*SLT1 Mean (sd)*	*SLT2 Mean (sd)*	p SLT1 vs SLT2	*p-value*
IOP	Baseline (*n* = 104,33)	20.0 (5.3)	18.8 (4.9)	0.02	21.3 (4.8)	21.1 (5.0)	0.84	0.22 0.02
	6–12 months (*n* = 102,92,28,27)	15.3 (3.3)	16.4 (4.8)	0.03	20.0 (5.4)	16.2 (5.1)	0.004	<0.001 0.82
	12–15 months (*n* = 97,80,13, 19)	16.3 (4.0)	16.9 (4.8)	0.19	19.3 (1.2)	16.1 (4.6)	0.58	0.19 0.50
IOP decrease	6–12 months	4.7 (4.6)**	2.4 (4.3)**	<0.001	1.9 (5.0)	4.8 (5.6)**	0.11	0.007 0.02
IOP decrease	12–15 months	3.6 (4.7)**	1.7 (4.1)**	0.004	0.3 (2.5)	4.2 (5.5)*	0.73	0.24 0.03
IOP % reduction	6–12 months	20.5 (18.5)**	12.4 (24.0)**	0.02	7.6 (21.2)	22.5 (23.7)**	0.05	0.002 0.07
IOP % reduction	12–15 months	15.0 (23.5)**	8.9 (20.9)**	0.09	0.2 (12.8)	19.4 (25.8)*	0.66	0.28 0.06

The effect of glaucoma medications was also studied. Patients were divided into those using prostaglandin analog (PGA) medications after laser and those not using them. There was a trend toward higher success rates in patients not using a PGA. The success of SLT 2 for patients using a PGA was 36 % at 6 months and 18 % at 12 months, versus 44 and 25 %, respectively, for those not using them. Using definition number two, the success rates were 47 and 24 % for PGA and 56 and 42 % for no PGA. These differences were not statistically significant when compared using the log rank test for survival curve comparison (*p* = 0.44, *p* = 0.22).

The rate of success for SLT2 was also stratified by whether SLT 1 met the success criteria, but there was no difference in the success of SLT 2 (survival curves not shown).

## Discussion

Sequential treatment with SLT after ALT has demonstrated success since the initial clinical study by Latina et al. [12]. The study included two SLT treatment arms: one with uncontrolled IOP on maximal medical therapy, and the second with uncontrolled IOP with prior failed ALT. The sequential treatment group (ALT then SLT) had a mean IOP reduction of 3.8 mmHg from a baseline of 25.3 mmHg, which was comparable to the group without any prior laser treatment.

The first published clinical study on the success of repeat SLT was by Hong et al. [23]. In this retrospective review, 44 eyes of 35 open-angle glaucoma patients with a prior 360-degree SLT that was successful for 6 months but then lost efficacy were treated with a repeat 360-degree SLT. The IOP was recorded prior to the procedure and 1–4 weeks, 1–3 months, and 5–8 months after treatment. The reduction in IOP after SLT 1 and SLT 2 was not statistically different at any time point, except at 1 to 3 months, when reduction was greater after SLT 1. Using a definition of success of at least a 20 % IOP reduction, the authors found no difference between SLT 1 and 2. They also found no difference whether the SLT was repeated within 6–12 months of SLT 1 versus after 12 months. They concluded that repeat 360-degree SLT is safe and effective after an initially successful 360-degree SLT has lost efficacy, and that this may be accomplished as early as 6 months after the initial laser.

Our study had a similar design with some notable exceptions. The number of subjects in our study was significantly greater, and our study had longer follow-up. We reported only data from one eye of each patient in order to reduce any bias. With the larger sample size, we were able to perform several subanalyses, including case-matching and analyzing the length of time between laser treatments and type of medication use. The multi-center design has the drawback of a non-standardized procedure and patient treatment protocol. However, it also allows for greater numbers of subjects and enhances the statistical analysis. Our study was retrospective rather than prospective, with the limitations inherent to this design such as investigator bias, non-standardization of treatment and data collection, and lack of a control group.

We elected to include patients that had prior ALT in our analysis. This scenario is becoming increasingly apparent in clinical practice, as many patients that had initial laser trabeculoplasty with ALT have also now had SLT followed by repeat SLT. Therefore, we included this group in order to extend the applicability of results to patients that had prior ALT, not just those with only SLT. We performed an analysis of the main outcome measures without this group, and did not see any difference in the reduction of IOP or in the comparison of IOP levels or reduction after either SLT1 or SLT2.

The success criteria applied for this study are quite stringent and are the same as those used to determine the success of incisional filtration surgery in the Tube versus Trabeculectomy Study [24]. We felt that this criteria was best because it is evidence-based and incorporates a percentage IOP decrease. Since laser trabeculoplasty is often used to reduce dependence on glaucoma medications rather than to eliminate the need for medications, we added the second success definition, which includes reduction of medications, while maintaining IOP, as a success. One limitation of medication reduction as an outcome measure (in the absence of a washout) is that it does not account for some of the medications having already lost efficacy prior to treatment.

The data from our study did support the findings of the initial repeat SLT study. Repeat 360-degree SLT resulted in a reduction of IOP to a similar level, although the magnitude of decrease was greater for SLT 1 due to a higher baseline. The differences in baseline likely reflect clinical practice, where a surgeon will generally not wait until the IOP has returned all the way to the initial pre-treatment baseline but will perform a second treatment when the IOP begins to rise. However, when patients with similar baselines were examined, the amounts of IOP lowering and success rates were very similar.

Interestingly, a shorter time period between the first and second SLT resulted in statistically significant higher success rates for the effect of a repeat laser. This was true for both definitions of success used. We had initially hypothesized that a longer time period between lasers indicated a more effective first treatment and would predict a greater success for the repeat SLT.

The analysis of medications used (using a PGA versus not) showed no significant difference. We hypothesized that there may be a greater effect of SLT and repeat SLT in those not using a PGA. This was due to the data from Alvarado et al. showing a similar mode of action of PGA drugs and SLT laser, with both exerting an effect on Schlemm’s canal endothelial cells [25, 26]. There was a small trend towards greater efficacy of SLT without PGA medications, and perhaps larger numbers of patients may show a difference. Our study was not powered to answer this specific question.

## Conclusions

In conclusion, selective laser trabeculoplasty is successful in the initial and repeat treatment of open-angle glaucomas. This scenario has included patients who have had an initial successful treatment with 360-degree SLT lasting at least 6 months, with the effect wearing off over time. A second application of 360-degree SLT showed successful lowering of IOP or medications in these cases. This is a distinct advantage over argon laser trabeculoplasty (ALT), in which repeated treatments result in progressive damage to the trabecular meshwork and diminishing success. The difference may be attributable to the selective targeting of pigmented cells with SLT and the lack of coagulative damage to the outflow tract.

## Abbreviations

ALT, argon laser trabeculoplasty; IOP, intraocular pressure; Nd:YAG, neodymium-doped yttrium aluminium garnet; OAG, open-angle glaucoma; PGA, prostaglandin analog
